# Regulatory T cells control endothelial chemokine production and migration of T cells into intestinal tumors of APC^min/+^ mice

**DOI:** 10.1007/s00262-018-2161-9

**Published:** 2018-04-18

**Authors:** Paulina Akeus, Louis Szeponik, Filip Ahlmanner, Patrik Sundström, Samuel Alsén, Bengt Gustavsson, Tim Sparwasser, Sukanya Raghavan, Marianne Quiding-Järbrink

**Affiliations:** 1 Department of Microbiology and Immunology, Institute of Biomedicine, The Sahlgrenska Academy, University of Gothenburg, Box 435, 405 30 Gothenburg, Sweden; 2 Department of Surgery, Institute of Clinical Sciences, the Sahlgrenska Academy, University of Gothenburg, Gothenburg, Sweden; 3 Centre for Experimental and Clinical Infection Research, Institute of Infection Immunology, Twincore, Hanover, Germany

**Keywords:** Regulatory T cells, CXCR3, APC^min/+^, Colon cancer, Migration

## Abstract

Tumor-infiltrating lymphocytes are crucial for anti-tumor immunity. We have previously shown that regulatory T cells (Treg) are able to reduce T-cell transendothelial migration in vitro and accumulation of effector T cells in intestinal tumors in vivo. Treg depletion also resulted in increased levels of the chemokines CXCL9 and CXCL10 specifically in the tumors. In this study, we investigated the mechanisms for Treg mediated suppression of T-cell migration into intestinal tumors in the APC^min/+^ mouse model. By breeding APC^min/+^ mice with DEREG mice, which harbour a high affinity diphtheria toxin receptor under the control of the FOXP3 promoter, we were able to deplete Treg in tumor-bearing mice. Using adoptive transfer experiments, we could document a markedly increased migration of T cells specifically into Treg depleted tumors, and that Treg depletion results in increased production of the CXCR3 ligand CXCL10 from endothelial cells in the tumors. Furthermore, we were able to demonstrate that T cells use CXCR3 to migrate into intestinal tumors. In addition, human colon adenocarcinomas express high levels of mRNA CXCR3 ligands and tumor endothelial cells produce CXCL9 and CXCL10 ex vivo. In conclusion, this study demonstrates that Treg reduce endothelial CXCL10 production, inhibit T-cell migration into tumors and that CXCR3 mediated signalling is crucial for lymphocyte accumulation in intestinal tumors. Thus, immunotherapy aimed at Treg depletion may be effective by increasing not only T effector cell activity, but also their accumulation in tumors.

**Electronic supplementary material:**

The online version of this article (10.1007/s00262-018-2161-9) contains supplementary material, which is available to authorized users.

## Introduction

Colorectal cancer (CRC) is the third most prevalent cancer form world-wide with approximately one million new cases diagnosed each year [1]. About 5–10% of all colorectal cancer develops from hereditary nonpolyposis colorectal cancer or familial adenomatous polyposis (FAP), while the large majority of cases are sporadic. However, both FAP and a majority of sporadic colorectal cancer are due to mutations in the tumor-suppressor gene adenomatous polyposis coli (*Apc*) [2, 3]. Loss of *Apc* will result in polyps in both humans and mice, which are caused by a constitutive wnt signalling resulting in a continuous β-catenin-initiated gene transcription [4, 5]. Although many of the mutations that give rise to colorectal tumors have been identified, growing evidence demonstrates that the immune system also plays an important role in reducing tumor progression and improving patient outcome. Tumor-infiltrating lymphocytes (TIL), like natural killer (NK) cells, CD8^+^ cytotoxic T cells and CD4^+^ T helper (Th) cells have all been found to promote anti-tumor immunity [2, 6]. Previous studies from both our group and others have demonstrated an accumulation of regulatory T cells (Treg) in both human [7–9] and mouse [10, 11] intestinal tumors. Treg can control TIL function [12], but their role in CRC progression is currently unclear. In some studies, intra-tumoral Treg appear to play a favourable role for patient survival, possibly by reducing intestinal inflammation [13, 14], while in other studies they correlate to a negative overall survival due to an inhibited TIL response [15]. Recently, Saito et al., have proposed a model with two different populations of CD4^+^FOXP3^+^ cells in CRC, suppressive FOXP3^high^ Treg and FOXP3^low^ non-suppressive effector T cells, and that the balance between the two subsets determine tumor progression [16]. In addition, the appearance of RORγt^+^ IL-17-expressing Treg in tumors may be particularly unfavourable, as they shift the Th1/Th17 balance to favour tumor progression [17, 18]. Thus, the full extent of Treg mediated immune suppression and its contribution to colon cancer progression is still not established.

Infiltration of immune cells into tissues is regulated by chemoattractant chemokines and adhesion molecules, which orchestrate the immune balance and trafficking of lymphocytes into inflamed tissue [19]. We recently showed that Treg depletion results in an increased accumulation of effector T cells in intestinal tumors. This observation was accompanied by an increased intra-tumoral expression of the chemokines CXCL9 and CXCL10 [20]. These chemokines are both ligands to the Th1 associated chemokine receptor CXCR3, which is mainly expressed on activated Th1 cells, cytotoxic T cells, NK cells and dendritic cells [21]. It is thus interesting to note that Treg depletion also led to increased frequencies of conventional T cells expressing CXCR3 in the tumors [20]. Several studies have also shown that CXCR3 expression on T cells, or expression of CXCL9 and CXCL10 in tumor tissue, is associated with increased TIL accumulation and a favourable clinical outcome in CRC [22–24].

In previous studies, we could demonstrate that Treg from cancer patients, but not healthy volunteers, inhibit transendothelial migration of effector T cells in vitro and that effector T cells accumulate in intestinal tumors in vivo after Treg depletion [20, 25]. In this study, our aim was to elucidate the mechanisms whereby Treg reduced the lymphocyte accumulation in tumors, with a focus on cell migration and chemokine signalling. The APC^min/+^ mouse is widely used to model CRC, as it has a mutation in the *Apc* gene, similar to FAP and sporadic human CRC [5]. These mice develop tumors along the entire intestine and can be used to study early events of CRC since this is a non-invasive, non-metastasising model [26]. However, immunologically the APC^min/+^ tumors mimic the human counterpart well since they both show accumulation of Treg, a shift in lymphocyte composition and changed chemokine expression compared to unaffected intestine [7, 9, 11, 27]. By breeding APC^min/+^ mice with depletion of regulatory T cell (DEREG) mice, which harbour a high affinity diphtheria toxin (DT) receptor under the control of the FOXP3 promoter [28], we were able to deplete Treg by DT injections in tumor-bearing mice. This model was used to investigate the effects of Treg on lymphocyte migration into tumors and chemokine production in tumors. We report that Treg depletion results in an increased migration of T cells specifically into tumors. Furthermore, CD4^+^ T cells depend heavily on CXCR3-mediated signalling to enter into intestinal tissues. As Treg were shown to reduce the expression of the CXCR3 ligand CXCL10 by blood vessel endothelial cells, we suggest that Treg reduce effector T-cell recruitment to tumors by acting on local tissue cells to inhibit chemokine production.

## Materials and methods

### Mouse strains and breeding

APC^min/+^ mice, on a C57BL/6 background and DEREG mice [28] were bred to generate APC^min/+^/DEREG mice and APC^min/+^ mice at the Department of Experimental Biomedicine, University of Gothenburg. 4 weeks after birth APC^min/+^ genotype was examined by PCR and DEREG phenotype by flow cytometry as previously described [28, 29]. Animals were kept under specific pathogen-free conditions in filter-top cages.

### In vivo Treg depletion

Treg were depleted in both female and male 18 week old APC^min/+^/DEREG mice by intraperitoneal (i.p) injections of 0.5 µg DT on day 1, 2, 8, and 9. As controls, APC^min/+^ mice were identically treated. By day 3 and 10, at least 90% of Treg were depleted in blood, intestine and tumors of DT treated APC^min/+^/DEREG [20]. Mice were killed and organs harvested on day 12.

### Mouse lymphocyte isolation

Lamina propria cells from small intestinal mouse tumors and unaffected intestine were isolated as previously described [11, 30]. Briefly, epithelial cells were removed by HBSS medium containing EDTA, FCS, and HEPES. The remaining lamina propria was digested with either collagenase D (Roche) for lymphocyte isolation or collagenase P (Sigma–Aldrich) and dispase II (Roche) for endothelial cell isolation at 37 °C. Single-cell suspension was prepared from spleen and mesenteric lymph nodes (MLN) by forcing the organs through nylon nets using a syringe plunger. Red cell lysis was performed on splenic cells with 0.07 M NH_4_Cl, pH 7.3, at 37 °C for 5 min.

### Adoptive transfer

MLN and splenic lymphocytes were harvested from female WT mice at age 10–14 weeks. MLN cells were subsequently stained with CFSE (Vybrant CFDA-SE; Molecular Probes) and cell labelling was confirmed by flow cytometry. A total of 25 million cells in a volume of 200 µL PBS were injected via the tail vein into DT treated APC^min/+^ and APC^min/+^/DEREG mice at day 10 after the first DT treatment.

In other experiments, splenic cells were treated with 1 µmol/L of the CXCR3 antagonist AMG487 (Tocris) for 6 h or left untreated as control. 25 millions cells from each group were either labelled with CellTrace FarRED (ThermoFischer) or CFSE, mixed 1:1 and co-injected via the tail vein into DT-treated APC^min/+^/DEREG mice and control APC^min/+^ mice. Labels were alternated in different experiments to rule out potential effects of labelling on cell migration.

2 days after adoptive transfer the mice were killed and MLN, spleen, small intestine and small intestinal tumors were collected and weighed. Lymphocytes were isolated as previously described and the frequencies of retrieved CFSE- and FarRED-labelled cells were determined by flow cytometry.

### Colon cancer patients, specimen collection and cell isolation

Patients were scheduled for curative surgery of primary colon adenocarcinoma at Sahlgrenska University Hospital, Gothenburg, Sweden (see supplementary table 1 for patient characteristics). A piece of colon tumor and unaffected colon mucosa was collected from colon cancer patients at the time of surgery. None of the patients had undergone radiotherapy or chemotherapy for at least 3 years prior to colectomy, and none suffered from autoimmune diseases. Tissue specimens were transported on ice and immediately used for cell isolation. Single cell suspensions were prepared mainly as previously described [31] with a modification of Liberase instead of collagenase for enzymatic digestion. Subsequently, cell suspensions were incubated 12 h at 37 °C; a protein transport inhibitor (BD Golgistop, BD) was added the last 4 h.

### Flow cytometry

Single cell suspensions from mouse experiments were stained with Live/dead Aqua (Molecular Probes, Inc.), CD4-PerCP, CD8-Pacific Blue, CD69-BV605, CD62E-PE, CD62E-BV421, CD62P-Alexa647, CD162-AlexaFlour647, HECA454-PE (BD Bioscience), CD25-APC, CD31-BV605, CD105-Pacblue, I/A-I/E-BV421, CD45-APCCy7, EpCAM-APC-Cy7, Podoplanin-PE, CD31-PECy7, EpCAM-FITC, CD64-BV711 (Biolegend), CD11c-PECy5.5 (Invitrogen) CXCR3-PECy7, CD3-Alexa700 and Ly6C-efluor450 (eBioscience). For subsequent detection of CXCL10 mRNA Primeflow^®^ RNA Assay (eBioscience) was used accordingly to manufacture protocol. Samples were acquired on an LSR-II flow cytometer (BD Biosciences) and analysed using FlowJo software (Tree Star Inc.).

Single cell suspension isolated from human tumors and unaffected colon tissue were stained with Live/dead Aqua (Molecular Probes, Inc.), CD31-Alexa700, (Biolegend), CD4-PerCP, CD8-BV711, CD105-APC, CD14-Alexa700, CD19-APCH7 and CD19-PE-CF594 (BD bioscience) followed by permeabilization with Fix & Perm kit (ADG Bio research GMBH) and staining with CXCL9-FITC (R&D) and CXCL10-PE (Biolegend), flow cytometry analyses were performed as above.

### RNA and DNA isolation and quantitative PCR

Total RNA was isolated and used for cDNA synthesis as previously described [11]. Each real-time PCR reaction mixture contained cDNA, Power SYBR Green master mix (Applied Biosystems) and oligonucleotide primers to detect CXCL9 (forward: 5′-GAC CTT AAA CAA TTT GCC CCA AG-3′, reverse: 5′-TCC TTC ACC CCC ATC TGC TGA ATC TGG-3′), CXCL10 (forward: 5′-GAA ATT ATT CCT GCA AGC CAA TTT-3′, reverse: 5′-TCA CCC TTC TTT TTC ATG TAG CA-3′), CXCL11 (forward: 5′-GCA GAT ATT GAG AAA GCC TCC A-3′, reverse: 5′-TGG GAT TTA GGC ATC GTT GT-3′) and 18S RNA (forward, 5′-CGG C-GT TAT TCC CAT GAC-3′, reverse, 5′-AAG TTT CAG CTT TGC AAC CA-3′). All primers were designed using Primer Express software (Applied Biosystems), and were all ordered from Eurofins MWG. Relative expression was determined by the ΔΔC_T_ method using 18S RNA as endogenous control.

### Statistical analysis

Calculation of statistical significance was performed using Mann–Whitney test and Wilcoxon signed rank test. *p* values of < 0.05 were considered significant. Statistical analyses were performed in GraphPad PRISM software version 6.0 (GraphPad Software Inc., San Diego, CA).

## Results

### T-cell migration to tumors after Treg depletion

**Fig. 1:**
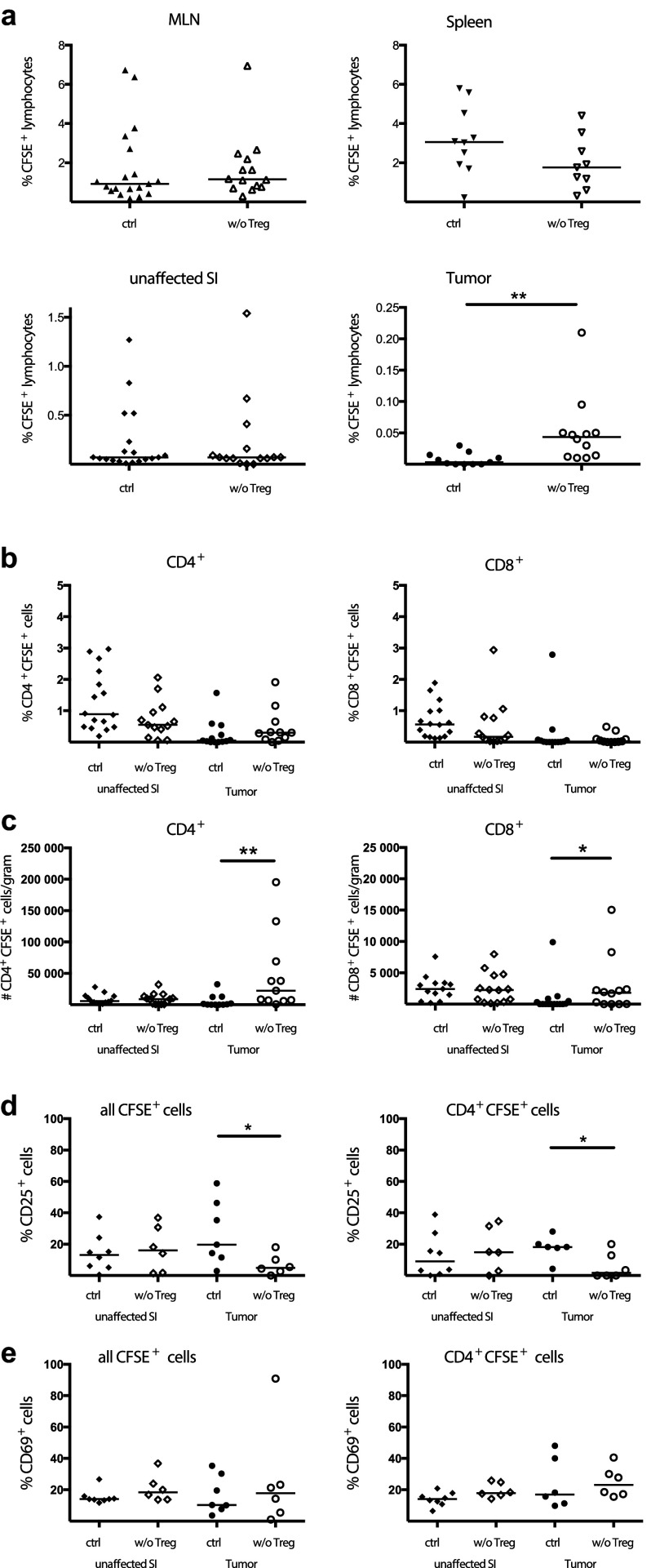
Increased migration into tumors after Treg depletion. CFSE-labelled MLN lymphocytes were transferred into DT treated APC^min/+^/DEREG and control APC^min/+^ mice. 2 days later MLN, spleen, unaffected small intestine (SI) and intestinal tumors were harvested and migrated CFSE-labelled cells were examined by flow cytometry. **a** Percentage of CFSE^+^ lymphocytes recovered after adoptive transfer among all lymphocytes in MLN, spleen, small intestine and tumor. **b** Percentage of CFSE^+^CD4^+^ and CFSE^+^CD8^+^ cells among all CD4^+^ and CD8^+^ cells, respectively, in small intestine and tumors. **c** Number of CFSE^+^ CD4^+^ and CFSE^+^CD8^+^ T cells per gram tissue recovered after adoptive transfer in small intestine and tumors. CD25 (**d**) and CD69 (**e**) expression among CFSE^+^ cells were characterized using flow cytometry both by all migrated CFSE^+^ lymphocytes and by all migrated CD4^+^CFSE^+^ cells. Symbols represent individual mice and lines represent median (*n* = 6–19). * *p* < 0.05, ** *p* < 0.01; Mann–Whitney test

Previously, we have shown an accumulation of TILs after Treg depletion in APC^min/+^ mice [20], although it was not clear to what extent this resulted from increased migration into the tumor tissue. As we previously demonstrated in vitro [25], Treg may inhibit T-cell accumulation by reducing transendothelial migration. To investigate if Treg affect migration into tumors in vivo, we depleted Treg in APC^min/+^/DEREG mice and transferred CFSE-labelled MLN cells and examined their migration compared to that in DT-treated control APC^min/+^ mice still harbouring Treg (see supplementary Fig. 1 for gating strategy). No significant differences in lymphocyte migration into MLN, spleen and unaffected small intestine were observed in Treg depleted APC^min/+^/DEREG mice compared to control mice (Fig. 1a). In contrast, there was a significant increase in the migration of transferred CFSE^+^ cells into the tumor tissue after Treg depletion in the APC^min/+^/DEREG mice (Fig. 1a), a finding that clearly confirms that Treg can reduce lymphocyte migration specifically into tumors. However, even though Treg were depleted and more effector T cells were recruited to the tumors, this short-term Treg depletion in mice with established tumors did not significantly affect tumor load (supplementary Fig. 2).

When the T-cell subsets among migrating cells were analysed, there was no difference in the frequencies of CFSE-labelled cells among CD4^+^ and CD8^+^ TILs with and without Treg (Fig. 1b). However, when analyzing the number of migrated cells per gram tissue, it was clear that both CD4^+^ T helper cell and CD8^+^ cytotoxic T-cell migration was significantly increased in the tumors after Treg depletion. There was a larger migration of CD4^+^ T cells, as higher numbers of transferred CD4^+^ T cells were observed in the tumors compared to CD8^+^ T cells (Fig. 1c). Presumably, many non-labelled T cells were recruited along with the adoptively transferred cells to the tumors, and therefore, the frequencies of CFSE^+^ cells among all CD4^+^ and CD8^+^ T cells did not change. To compare the composition of cells migrating into Treg depleted and Treg sufficient tumors, adoptively transferred CFSE^+^ lymphocytes as well as the CFSE^+^CD4^+^ T cells subset, were investigated for their expression of the mouse Treg marker CD25 [32, 33] and the activation marker CD69 (Fig. 1d–e). A significant decrease in migrating CD25^+^ lymphocytes was observed in the absence of Treg in tumors of the recipient, both among CD4^+^ T cells and all CFSE^+^ cells (Fig. 1d). In contrast, recently activated lymphocytes expressing CD69 migrated equally well into unaffected small intestine and tumors, regardless of the presence of Treg (Fig. 1e). Taken together, these results show that Treg depletion in APC^min/+^ mice result in a specific recruitment of CD4^+^ and CD8^+^ T cells without the Treg marker CD25 into tumors, but not to other organs.

### Selectin expression after Treg depletion

**Fig. 2:**
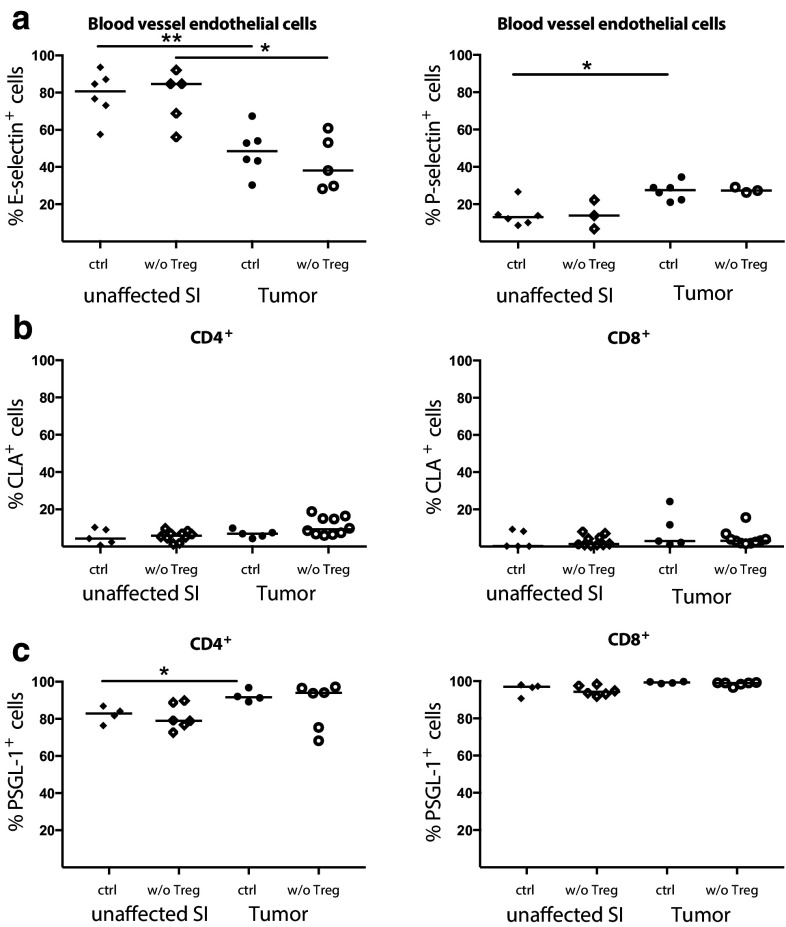
Expression of P- and E-selectin and their ligands in Treg depleted tumors. **a** Percentage of E-selectin^+^ and P-selectin^+^ among blood vessel endothelial cells (CD31^+^Podoplanin^−^) and CLA^+^ (**b**) and PSGL-1^+^ (**c**) expression among CD4^+^ and CD8^+^ T cells in Treg depleted APC^min/+^/DEREG and control APC^min/+^ mice. Symbols represent individual mice and lines represent median (*n* = 3–10). * *p* < 0.05, ** *p* < 0.01; Mann–Whitney test

Selectins play an important role in lymphocyte migration into tissue during inflammation and altered selectin expression might be one reason for the increased T-cell migration after Treg depletion. Using flow cytometry analyses of CD31^+^Podoplanin^−^ blood vessel endothelial cells, we could show that both P- and E-selectin are expressed by blood vessel endothelial cells at moderate frequencies in tumor tissue. However, both P- and E-selectin expression was unchanged after Treg depletion (Fig. 2a). Interestingly, selectin expression in tumors differed from unaffected small intestinal tissue. E-selectin was expressed at significantly lower levels in tumors than unaffected tissue, regardless of Treg depletion. Tissue-infiltrating lymphocytes were stained with antibodies to CD162, also known as P-selectin Glycoprotein Ligand-1 (PSGL-1), which preferentially recognize the P-selectin ligand, and to Cutaneous Lymphocyte Antigen (CLA), a modified PSGL-1 preferentially binding to E-selectin and identified with antibody clone HECA-452. Both P-selectin expression on tumor endothelial cells and PSGL-1^+^ expression on tumor-infiltrating CD4^+^ T cells were significantly higher compared to small intestine (Fig. 2a, c) suggesting that this pathway may contribute to T-cell recruitment into intestinal tumors. However, there were no significant differences in selectin ligand expression on tumor-infiltrating T cells with and without Treg (Fig. 2b, c), and therefore other mechanisms of T-cell recruitment are probably more important after Treg depletion.

### Lymphocyte migration into tumors after CXCR3-blocking

**Fig. 3:**
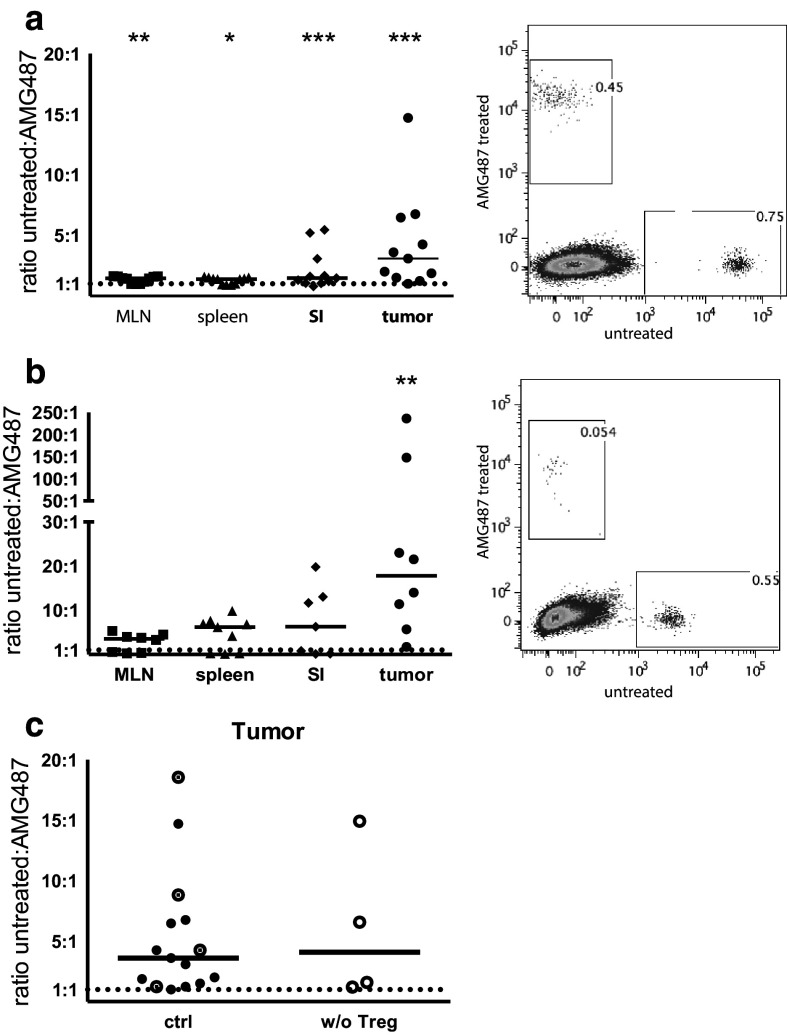
Reduced lymphocyte migration into tumors after CXCR3 blocking. Splenic lymphocytes were treated with AMG487 or left untreated for 6 h before labelling with CFSE or FARred. Equal numbers of both cell suspensions were injected into APC^min/+^ mice and 2 days later MLN, spleen, unaffected small intestine and intestinal tumors were harvested and the ratio between migrated CFSE- and FARred-labelled cells were determined by flow cytometry. **a** Ratio of untreated to AMG487-treated lymphocytes recovered from the indicated tissues. Dot plot show a representative analysis of untreated vs. AMG487 treated lymphocytes migrating into a tumor. **b** Ratio of untreated to AMG487-treated CD4^+^ T cells recovered from the indicated tissues. Dot plot show a representative analysis of untreated vs. AMG487 treated CD4^+^ T cells migrating into a tumor. **c** Ratio of untreated to AMG487-treated lymphocytes recovered in Treg depleted and control tumors. Circles with a dot represent DT treated control mice and filled circles untreated control mice from **a** for comparison. Symbols represent individual mice and lines represent median. * *p* < 0.05, ** *p* < 0.01, *** *p* < 0.001 compared to the expected 1:1 ratio; Wilcoxon signed rank test (*n* = 4–15). Dotted line represents a hypothetical uniform migration at 1:1 ratio

Chemokines guide lymphocytes into tissue and increased chemokine production might also be a reason for the increased migration into tumors. Our previous study [20] indicated that CXCR3-mediated chemotaxis may be an important means of T-cell recruitment into intestinal tumors as Treg depletion increased the production of CXCL9 and CXCL10 selectively in the tumors. We thus examined the effect of CXCR3 blocking on migration of splenic lymphocytes into APC^min/+^ tumors in the adoptive transfer system. Pre-treatment of lymphocytes with the specific CXCR3 antagonist AMG487 [34] before transfer potently reduced migration into both unaffected small intestine and tumors with a median 3.1-fold higher migration of untreated cells compared to AMG487 treated cells in the tumors and a 1.5-fold increase in the unaffected tissue (Fig. 3a). On the other hand, migration of CXCR3-blocked lymphocytes into MLN and spleen was only slightly decreased. CD4^+^ T cells had a larger migration disadvantage into tumors when treated with the CXCR3 antagonist compared to all other tested organs (Fig. 3b). However, Treg depletion did not further increase the preferred migration of CXCR3^+^ lymphocytes into tumors (Fig. 3c). Taken together, these observations complement our previous observation of increased CXCR3 ligand expression in tumors after Treg depletion, and underline the specific importance of CXCR3 in lymphocyte, and more specifically CD4^+^ T cell, migration into intestinal tumors.

### Identification of CXCL10-expressing cells after Treg depletion

**Fig. 4:**
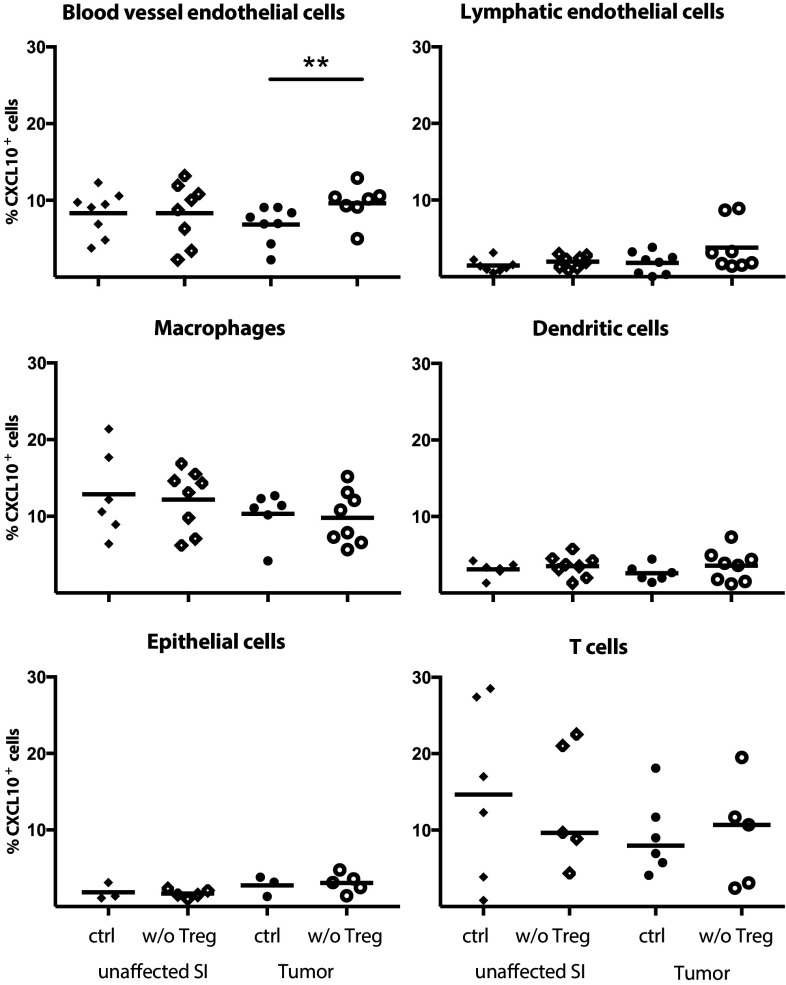
Increased blood vessel endothelial CXCL10 expression after Treg depletion. Frequencies of CXCL10^+^ cells among blood vessel endothelial cells (CD31^+^Podoplanin^−^), lymphatic endothelial cells (CD31^+^Podoplanin^+^), macrophages (MHCII^+^CD64^+^), dendritic cells (MHCII^+^CD11c^+^), epithelial cells (EpCAM^+^) and T cells (CD3^+^) in Treg depleted APC^min/+^/DEREG and control APC^min/+^ mice was investigated by Primeflow^®^ RNA Assay followed by conventional flow cytometry in unstimulated cells. Symbols represent individual mice and lines represent median (*n* = 3–8). ** *p* < 0.01; Mann–Whitney test

**Fig. 5:**
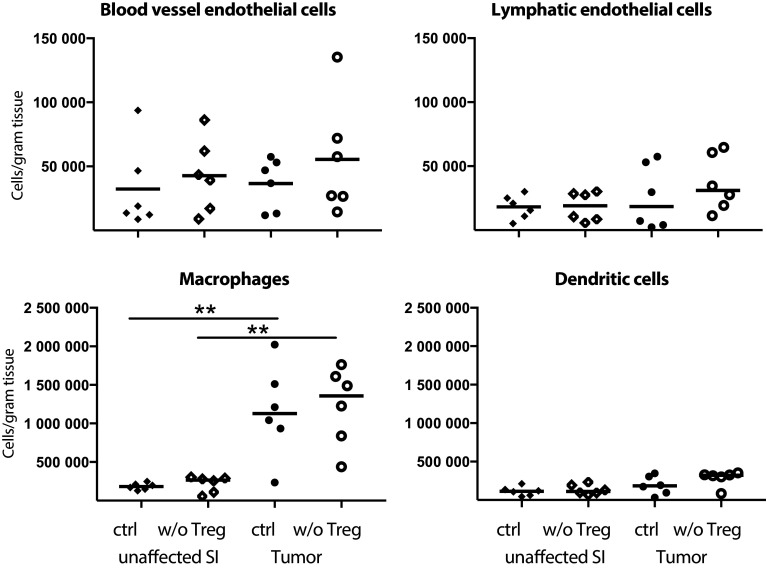
Accumulation of macrophages in intestinal tumors. Number of cells per gram tissue of blood endothelial cells (CD31^+^Podoplanin^−^), lymphatic endothelial cells (CD31^+^Podoplanin^+^), macrophages (MHCII^+^CD64^+^) and dendritic cells (MHCII^+^CD11c^+^) in Treg depleted APC^min/+^/DEREG and control APC^min/+^ mice investigated by flow cytometry. Symbols represent individual mice and lines represent median (*n* = 6). ** *p* < 0.01; Mann–Whitney test

As CXCR3-mediated recruitment appears crucial for T cell entry into tumors and as Treg reduce CXCL9 and CXCL10 expression in tumors, we were eager to determine the source of CXCL10 in tumor tissue, and determine if CXCL10-producing cells were indeed affected by Treg depletion. Single cell suspensions from unaffected small intestine and tumors were analysed for CXCL10-expressing cells ex vivo without any stimulation using Primeflow^®^ mRNA assay to detect intracellular RNA. CD31^+^Podoplanin^−^ blood vessel endothelial cells, CD31^+^Podoplanin^+^ lymphatic endothelial cells, MHCII^+^CD64^+^CD11c^−^ macrophages, MHCII^+^CD11c^+^CD64^−^ dendritic cells, EpCAM^+^ epithelial cells and CD3^+^ T cells all produce CXCL10 (Fig. 4, for gating see supplementary Fig. 3). When Treg were depleted, only blood vessel endothelial cells in the tumors significantly increased their production of CXCL10, indicating that Treg affect blood vessel endothelial cells to reduce recruitment of CXCR3^+^ effector lymphocytes. Macrophages, an important regulator of tumor progression [35], are known to produce CXCL10, and our data show that they do it to approximately the same extent as blood vessel endothelial cells but that macrophage CXCL10-production is unaffected by Treg depletion (Fig. 4). Neither blood vessel endothelial cell nor macrophage density changed after Treg depletion (Fig. 5). However, both density (Fig. 5) and frequencies (supplementary Fig. 4) of macrophages were higher in tumor tissue than unaffected small intestine, whereas no such increase was evident in any of the other cell types analysed. Taken together, our data suggest that both macrophages and blood vessel endothelia cells are important sources of CXCL10 in APC^min/+^ tumors.

### Production of CXCL9 and CXCL10 in human colon tumors

**Fig. 6:**
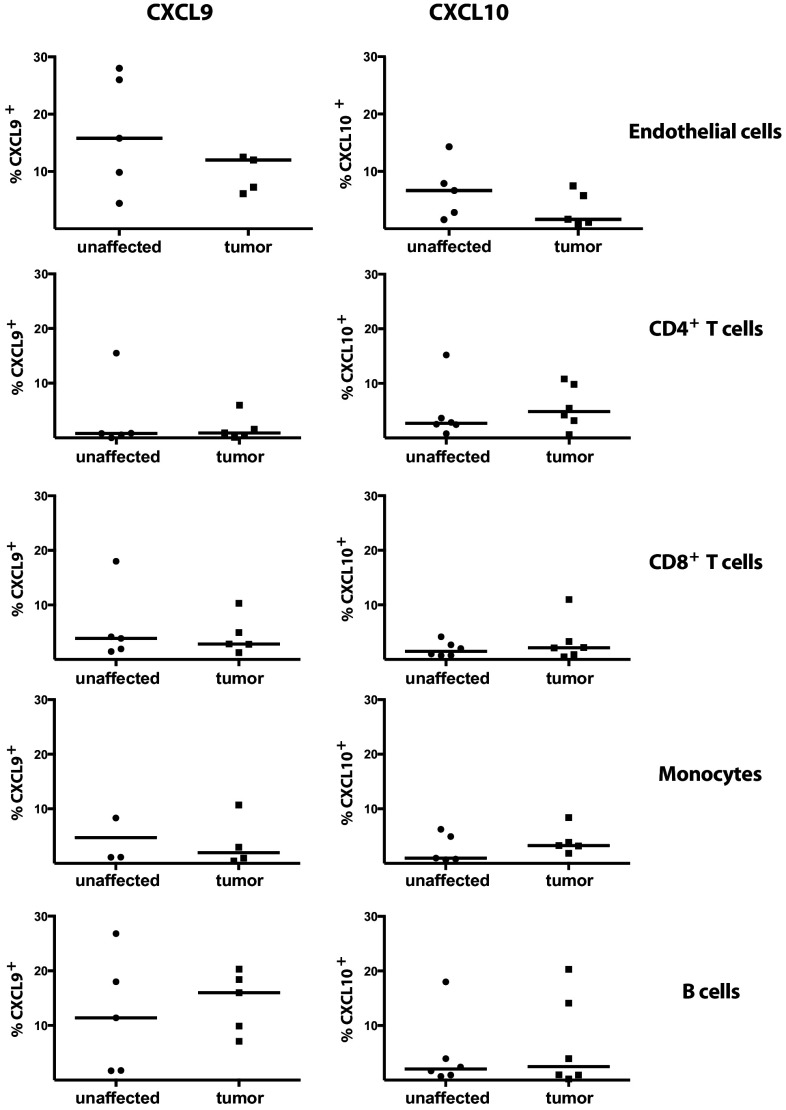
Production of CXCL9 and CXCL10 by cells in human colon cancer. Single cell suspension was prepared from tumors and unaffected human colon tissue and the frequencies of CXCL9- and CXCl10-producing, CD4^+^ T cells, CD8^+^ T cells, CD14^+^ monocytes, CD31^+^CD105^+^ endothelial cells and CD19^+^ B cells were determined by flow cytometry. Symbols represent individual patient and lines represent median (*n* = 3–6)

To determine the relevance of these findings in human tumors, we examined CXCR3 ligand production in human colon tumors. Initial quantitative PCR studies of ex vivo tissue specimen showed that CXCL9, CXCL10 and CXCL11 are significantly increased in human colon tumors compared to unaffected tissue (supplementary Fig. 5). To further investigate these chemokines in human CRC, we characterized the cells producing CXCL9 and 10 ex vivo without any stimulation, and found that human endothelial cells (defined as CD31^+^CD105^+^ cells) in colon tumors produce both chemokines (Fig. 6). In addition, CXCL9 and CXCL10 were also produced by T cells, monocytes and B cells.

## Discussion

Treg depletion increases lymphocyte frequencies in tumors [20, 36–38], and we now clearly demonstrate in a spontaneous tumor model that Treg in tumor-bearing mice inhibit lymphocyte migration specifically into tumors. Adoptively transferred CFSE-labelled lymphocytes were able to migrate into MLN, spleen, intestine and intestinal tumors, but when Treg were depleted, a significant increase of migration was observed only into the tumors. This study demonstrates unequivocally that lymphocyte accumulation in tumors after Treg depletion is due to an increased migration into tumors, and not only from an increased intra-tumoral proliferation or survival of effector T cells. In our current study, both CD4^+^ and CD8^+^ TILs were increased after Treg depletion. Using CD25 as a marker for Treg [32, 33, 39] we could also show that the increase in CD4^+^ T cells is not due to adoptively transferred Tregs re-populating the empty Treg niche, instead a decreased Treg migration into Treg depleted tumors was observed. This finding would indicate that in an untreated tumor, Treg already present act to recruit more Treg, thereby reinforcing an immunoregulatory microenvironment. Our previous study also identified a higher proportion of proliferating T cells in Treg depleted tumors [20] and this probably also contribute to the total increase in tumor-associated effector lymphocytes in the absence of Treg. In addition to increased proliferation of TIL when Treg were depleted, there was also an increased frequency of CD69^+^ recently activated or tissue resident memory T cells in tumors [20]. However, no difference in the CD69 expression of the migrating cells were found in Treg depleted mice compared to control mice and recently migrated cells had lower CD69 expression than tumor-resident T cells. This would indicate that activation of T cells may occur locally in the tumors by intra-tumoral antigen-presenting cells, and not only in the draining lymph nodes. Even though CD4^+^ and CD8^+^ T cells in the tumors express similar and high levels of chemokine receptors important for intestinal migration [20] higher numbers of CD4^+^ T cells migrate into the tumor. Apart from chemokines, adhesion molecules are also important for migration across the endothelial border of peripheral organs and inflamed tissue [19]. Higher levels of functional selectin ligands on Th1 cells than Th2 cells have been linked to a difference in migration into inflammatory sites [40] and similar selectin ligand differences have also been observed among CD4^+^ and CD8^+^ T cells migrating into sites of skin infection [41]. In our APC^min/+^ model, however, no difference in expression of the E-and P-selectin ligands was found on the tumor-infiltrating T cells after Treg depletion. Furthermore, no difference in E- and P-selectin expression was observed on blood vessel endothelial cells after Treg depletion, indicating that other mechanism of T-cell recruitment than selectin/selectin ligand interactions may be more important in the absence of Treg.

Previous studies have proposed that Treg depletion can promote high endothelial venule neogenesis and normalization of tumor vascularity, thereby promoting increased T-cell infiltration [36, 37]. However, we have not observed changes in vascularisation in our short-term depletion experiments using a spontaneous tumor model [20], and other mechanisms for increased migration might be more important in our system. We previously identified one possible mechanism for the increased accumulation of TILs in Treg depleted mice since they harbour conventional T cells with an increased expression of the chemokine receptor CXCR3 [20], which is preferentially expressed on Th1 cells and CD8^+^ cytotoxic T cells [42]. To further evaluate the importance of CXCR3 in migration to tumors, we used AMG487, a small molecule CXCR3 antagonist, which has been shown to inhibit Th1 cell migration into sites of inflammation [43, 44]. In this study, AMG487 treatment significantly reduced lymphocyte migration into both tumors and unaffected small intestine, with the largest difference seen in the tumors. In MLN and spleen, only a slight reduction of migration was observed, and this observation is in line with previous studies showing that CXCR3 is not necessary for migration into the spleen [45]. Furthermore, a particular importance of CXCR3 for migration of CD4^+^ T cells into tumors was observed since AMG487 treated CD4^+^ cells almost completely failed to migrate into tumors. This is the first time CXCR3 has been shown to be non-redundant for CD4^+^ T-cell migration into intestinal tumors, even though earlier results have indicated that CXCL9 and CXCL10 significantly correlate to disease free survival of CRC patients, and also to the recruitment of memory T cells [22]. Treg depletion experiments showed an equally preferred migration of CXCR3^+^ lymphocytes into tumors in treated as well as in control mice, indicating that there is no additional T cell recruiting mechanism acting in parallel to CXCR3-mediated migration in the Treg-depleted tumors. Woods et al. [46] recently showed that CXCL9 is expressed in tumor endothelial cells regardless of anatomical tumor location, and thus the effect of Treg that we identified in intestinal tumors might be generalized to other tumor types as well.

To investigate how Treg affect CXCR3-mediated migration we determined which cells express the CXCR3 ligand CXCL10 in the tumors. In line with previous studies [21], we found a broad range of cells with CXCL10 expression in the tumors. However, when examining CXCL10 expression in Treg depleted mice, only blood vessel endothelial cells specifically increased their CXCL10 production after Treg depletion. Although macrophages are clearly important for tumor progression [35] and also accumulate in the APC^min/+^ tumors, this finding would indicate that Treg primarily reduce CXCL10 production by blood vessel endothelial cells to avoid a Th1 type response. One may think that the increase in CXCL10 expressing blood vessel endothelial cells is relatively modest and may not be sufficient to mediate the increased recruitment of T cells demonstrated here. However, if endothelial cells themselves produce the chemokine, a large part of produced protein will presumably be available to migrating lymphocytes, compared to when the chemokine is produced by underlying tissue cells such as macrophages and has to be endocytosed by endothelial cells and transported to the apical surface. Furthermore, small changes in chemokine production can lead to large shifts in immune cell accumulation. This is demonstrated by studies of recruitment of IgA-producing plasmablasts to *Helicobacter pylori* infected gastric mucosa. When healthy and infected tissue was compared, the expression of MAdCAM-1 and CCL25 was similar, while CCL28 production was about doubled. Still, the recruitment of integrin α437^+^CCR9^+^CCR10^+^ IgA-secreting cells to the infected tissue was on average 80-fold larger than the to uninfected stomach [47, 48].

The accumulation of Treg in human colon cancer and adenomatous polyps is well reflected in the APC^Min/+^ mice [7–11]. In addition, human Treg also inhibit T cell transendothelial migration [12]. However, in mice there is only one isoform of CXCR3, while three different isoforms have been identified in humans (20). Furthermore, C57bl/6 mice lack CXCL11 due to a point mutation [49]. Consequently, we investigated if our chemokine data from the mouse model would correspond to human CRC, and found an increased expression of CXCL9, 10 and 11 in tumors compared to unaffected small intestinal tissue. Endothelial cells in human specimen were able to produce CXCL9 and CXCL10 in both unaffected tissue and tumor, confirming an endothelial origin of these chemokines in both species. Apart from mediating recruitment of protective immune cells, CXCL10 has been shown to reduce tumor progression independently of CXCR3. To this end, CXCL10 both increase the apoptotic rate of cancer cells [50] and inhibit endothelial cell proliferation [51]. Thus, Treg-mediated down-regulation of CXCL10 specifically by endothelial cells might give an advantage for tumors not only by avoiding an immune attack, but also by increasing angiogenesis.

In conclusion, we have shown that CXCR3 signalling is crucial for T-cell migration into intestinal tumors. Furthermore, Treg depletion in tumor-bearing mice increased production of CXCR3 ligands specifically in tumor blood vessel endothelial cells, and led to an increased T-cell migration into tumors. Thus, in addition to improved effector T-cell activity, another mechanism behind successful Treg-targeted immunotherapy may be to enhance the levels of CXCR3 ligands locally available in tumors, which in turn might increase the recruitment of cells with potent anti-tumor effector functions.

### Electronic supplementary material

Below is the link to the electronic supplementary material.

Supplementary material 1 (PDF 443 KB)

## Abbreviations

APC: Adenomatous polyposis coli
CLA: Cutaneous lymphocyte antigen
CRC: Colorectal cancer
DEREG: Depletion of regulatory T cell
DT: Diphtheria toxin
FAP: Familial adenomatous polyposis
MLN: Mesenteric lymph nodes
PSGL-1: P-selectin glycoprotein ligand-1
SI: Small intestine
