# Supplementary material for: Regulatory T cells control endothelial chemokine production and migration of T cells into intestinal tumors of APCmin/+ mice
Source: Cancer Immunol Immunother. 2018 Apr 18;67(7):1067–77. doi: 10.1007/s00262-018-2161-9 (PMC6006230; doi:10.1007/s00262-018-2161-9)
Supplement: Supplementary file 1 — Supplementary material 1 (PDF 443 KB) [file 262_2018_2161_MOESM1_ESM.pdf]

Supplementary figures

Supplementary table 1. Characteristics of the colon cancer patients included in the study.

		females	males
	n	17	24
	age	52-89	42-89
Tumor location	ascending	7	13
	transverse	5	5
	descending	5	6
Differentiation grade	high	3	2
	medium	11	19
	low	3	2
	mucinous	-	1
Tumor stage	T1	-	1
	T2	-	2
	T3	12	14
	T4	5	7
Lymph node spread		11	12
Distant metastases		1	1

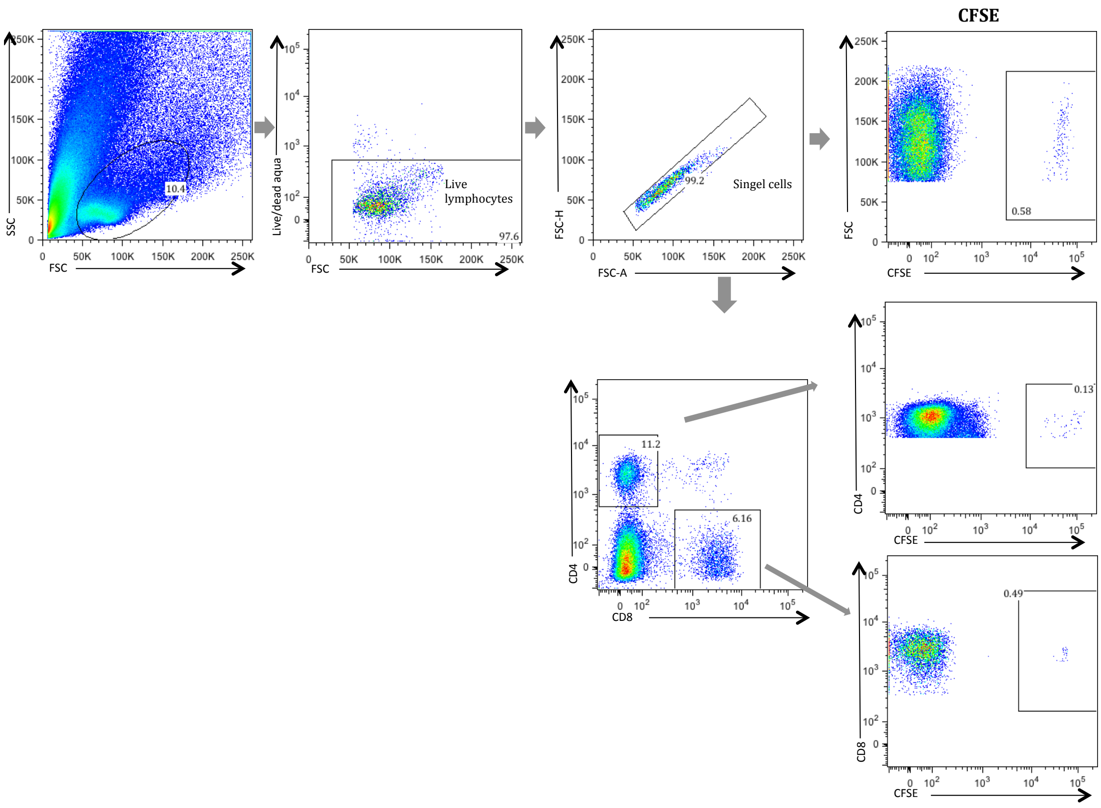

Supplementary figure 1: Gating strategy to detect migrating cells.
Gating strategy and representative flow cytometry plots showing CFSE expression among live single cells lymphocytes, $CD4^+$ and $CD8^+$ T cells in small intestinal tumor tissue from $APC^{min/+}$ mice after adoptive transfer.

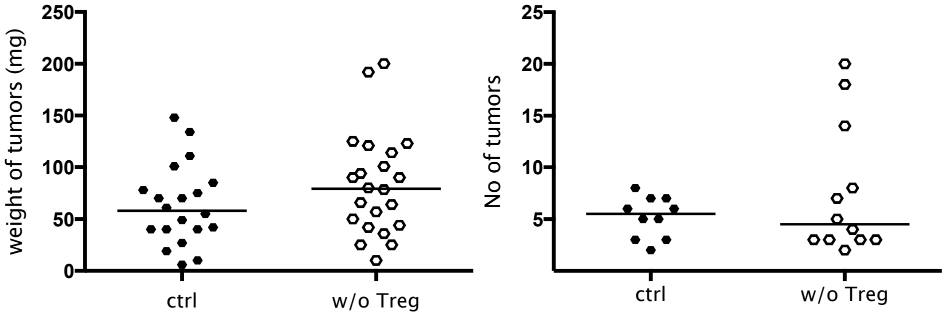

Supplementary figure 2: Unchanged tumor load after short-term Treg depletion.
DT treated 18-week old APC$^{min/+}$/DEREG mice and control APC$^{min/+}$ mice were sacrificed and small intestinal tumor tissue was collected and weighted. Symbols represent individual mice and lines represent median (n=10-21).

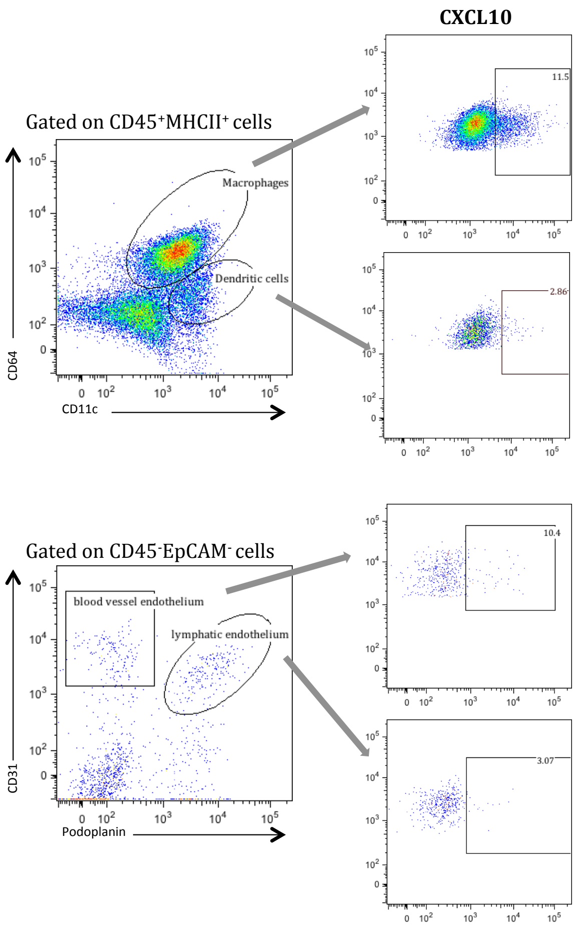

Supplementary figure 3: CXL10 expression in intestinal tumors.
Representative flow cytometry plots showing CXCL10 expression among macrophages (MHCII$^+$CD64$^+$), dendritic cells (MHCII$^+$CD11b$^+$), blood vessel endothelial cells (CD31$^+$Podoplanin$^-$) and lymphatic endothelial cells (CD31$^+$Podoplanin$^+$) in small intestinal tumor tissue from DT-treated APC$^{min/+}$/DEREG mice.

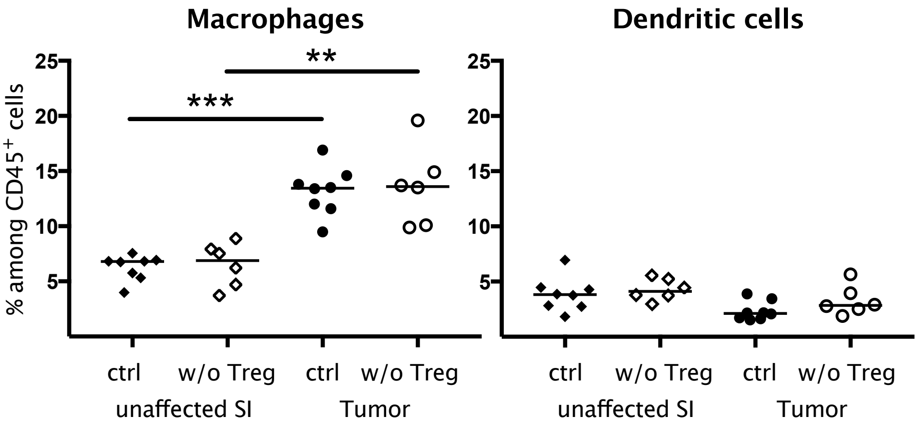

Supplementary figure 4: Macrophage and dendritic cell frequencies in intestinal tumors.

Frequencies of macrophages ($MHCII^{+}CD64^{+}$) and dendritic cells ($MHCII^{+}CD11b^{+}$) in Treg depleted $APC^{min/+}$/DEREG and control $APC^{min/+}$ mice were investigated by flow cytometry. Symbols represent individual mice and lines represent median (n=6-8). $^{**}p < 0.01$, $^{***}p < 0.001$; Mann-Whitney

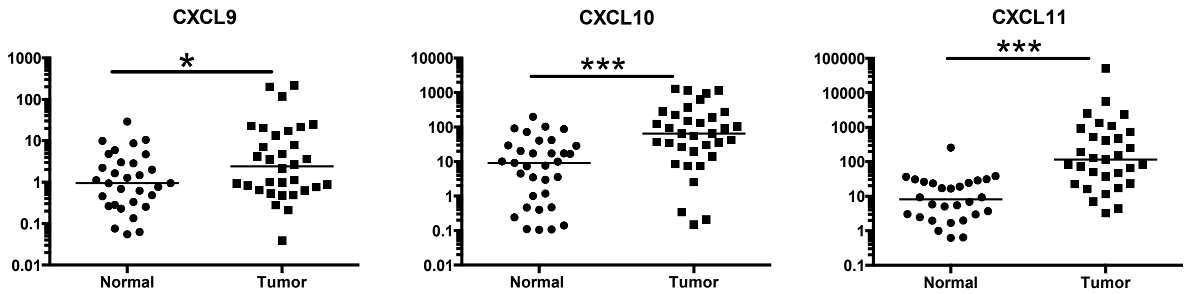

Supplementary figure 5: High expression of CXCR3 ligands in human colon tumors.

Tumor tissue and paired unaffected colon tissue from patient with colon adenocarcinoma were examined for expression of CXCL9, CXCL10 and CXCL10 mRNA by quantitative real-time PCR normalized against 18s. Symbols represent individual patients and lines represent median (n=27-31). $^{*}p < 0.05$, $^{***}p < 0.001$; Mann-Whitney.
